# On an Epidemic of Icterus in Roumania in 1917

**Published:** 1919

**Authors:** 


***On an Epidemic of Icterus in Roumania in 1917.*** By Dr. Z. Cantacuzene. *Presse Médicale,* Oct. 24, 1918.

The first cases of this epidemic were observed in May, 1917, and the incidence was chiefly confined to the army. Only in August did the civilian population get infected, and even then in infinitely smaller numbers.

The clinical and microbiological observations of the Roumanian doctors were very much hampered by the intensive work caused by other more virulent epidemics. However, it was possible to bring out a relatively clear pathological picture, and to see that this form of icterus differed from the previously known forms — recurring fever with icteric form, atypic typhoids with Eberth bacilli revealed by hemoculture and complicated by icterus, pseudo-classical paratyphoids often complicated with jaundice, dysentery form and gastro-intestinal states without dysenteric bacilli, sometimes complicated with subicterus.

During the six months preceding the epidemic, the Roumanian Army had had to undergo most trying physical conditions: an outbreak of cholera, which happily was soon checked, the disastrous retreat, exhausted regiments falling by the road-side, and dying of starvation, cold and fatigue. In January there was a veritable "syndrome of inanition" with cachectic state, edemas, non-specific dysentery, which nothing could stop, and such a state of weakness that secondary infections (abscesses, pleurisies, etc.) did not even provoke an increase of temperature.

The men could not tolerate any food and died in large numbers in the regimental hospitals. It was in this Army of Ghosts and Specters that an exanthemic typhus occurred in January, 1917, with recurring fever.

The feeding of the troops was absolutely insufficient; the flour was generally damaged. By that time the antityphic sera had lost a great deal of their protective powers and immunity was much diminished.

Although the epidemic was of a benign type, the incidence was exceedingly high. It was seen that a soldier on leave contamined first his family and finally the whole village.

An icteric man, sleeping in a barn for one night, gave icterus to eight other men sleeping in the same barn. It seems that man is the carrier of the infection. Observations do not tend to attribute contagion to water or to flies, which were very scarce in the mountains where the troops were stationed, nor to parasites.

Incubation takes from four to nine days. Patients report at first shivers and slight pyrexia, afterwards nausea and vomiting; later on they complain of tenderness in the vesicular region. On examination the liver is found enlarged and conjunctival, icterus appearing on the fourth and fifth day. Asthenia is very pronounced, constipation is frequent, the pulse is slow, and the spleen is also found enlarged.

The infection lasts from a week to a fortnight. Mortality is practically nil, save, however, for pregnant women. There it is of exceptional gravity and means death with almost certainty. At necropsy, extensive, fatty, degenerescence of the liver, and severe lesions of the surrenal capsules are found; sometimes the liver is one mass of fat.

The hepatic cells are filled with fat, and a large number of cells have disappeared under the invasion of the Kupfer cells and free macrophages. The very large distension of the intralobulary bile canalicules is the most striking feature. They are very dilated, full of stagnant bile, and obturated by agglomeration of leukocytes, the presence of which is in all probability the mechanical cause of the biliary stasis. The leukocyte thrombus is met only in the intralobular canaliculi.

The cytological study of the blood in the course of the infection does not show any excessive hyperleukocytosis; towards the end the globulary resistance has become almost normal.

None of the repeated and varied microscopic examinations have ever revealed any spirochetes in the blood, the urines, or in any organism of the system.

Generally speaking there are very few chances to isolate micro-organisms in hemoculture out of the febrile period, and the latter are very short indeed. It was possible to obtain nothing but paratyphic germs. In three cases the blood cultures remained negative; finally in two cases of serious icterus, laparatomized with a view to draining the bile vesicule, culture in the bile remained negative. On the whole, these micro-organisms present the following characters : mobile bacilli not stained by Gram, which do not liquify gelatin, while they produce on gelose bluish, transparent, and slightly mucous colonies.

The anti-para B sera have a very marked agglutinative power, varying from 1/500 to 1/2000 and even more.

When studying this agglutinative power it is important to differentiate between patients who have been vaccinated recently and those who have not antitypho-paratyphic vaccination; that is to say, if the patient has been vaccinated within the previous two months, the serum is seen to lose its entire agglutinative power towards the Eberth or the A and B paratyphics at the beginning of the icterus. This agglutinative power reappears after a week.

From various considerations as to the degree of agglutinative power, it is likely that these aberrant paratyphics are the pathogenous agents of the epidemic icterus. Out of eight infected cases only one underwent a classical syndrome of medium intensity; six had a light syndrome, and one had just a rough infection without icterus.

Vaccination may provoke a new attack, lasting often longer than the first one. It seems incomprehensible that the disease hardly affected the Russian Army living in contact with the Roumanian, though the former was far from having been totally vaccinated. The same observation was made by Clunet regarding the Dardanellès epidemic where the Allied troups suffered much more than the Turks, whose sanitary conditions were deplorable.

Could one deduct, then, that the B paratyphics are susceptible of modifying their primary characters, and constitute an autonomous, icterogenous race, capable also of giving birth to an aberrant clinical type?

